# Serum Level of Antibody against Benzo[a]pyrene-7,8-diol-9,10-epoxide-DNA Adducts in People Dermally Exposed to PAHs

**DOI:** 10.1155/2014/834389

**Published:** 2014-04-13

**Authors:** Lenka Borska, Ctirad Andrys, Jan Krejsek, Vladimir Palicka, Marcela Chmelarova, Kvetoslava Hamakova, Jan Kremlacek, Pavel Borsky, Zdenek Fiala

**Affiliations:** ^1^Institute of Pathological Physiology, Charles University in Prague, Faculty of Medicine in Hradec Kralove, 50038 Hradec Kralove, Czech Republic; ^2^Institute of Clinical Immunology and Allergology, Charles University in Prague, Faculty of Medicine in Hradec Kralove, 50038 Hradec Kralove, Czech Republic; ^3^Institute of Clinical Biochemistry and Diagnosis, Charles University in Prague, Faculty of Medicine in Hradec Kralove, 50038 Hradec Kralove, Czech Republic; ^4^Clinic of Dermal and Venereal Diseases, University Hospital Hradec Kralove, 50005 Hradec Kralove, Czech Republic; ^5^Institute of Hygiene and Preventive Medicine, Charles University in Prague, Faculty of Medicine in Hradec Kralove, 50038 Hradec Kralove, Czech Republic

## Abstract

Some specific antibodies indicate the presence of antigenic structures on DNA (DNA adducts) that can play an important role in the process of mutagenesis and/or carcinogenesis. They indicate the presence of increased genotoxic potential (hazard) prior to the formation of disease (primary prevention). The present study was focused on the serum level of benzo[a]pyrene 7,8-diol-9,10-epoxide-DNA adducts antibodies (anti-BPDE-DNA) in psoriatic patients (*n* = 55) dermally exposed to different levels of polycyclic aromatic hydrocarbons (PAHs). The general goal of the study was to contribute to better understanding of the value of the assumed biomarker (anti-BPDE-DNA) for evaluation of the organism's answer to genotoxic exposure to PAHs. Elevated level of exposure to PAHs resulted in the increased level of anti-BPDE-DNA. However, almost all levels of anti-BPDE-DNA ranged within the field of low values. Both variants of GT (CCT-3% and CCT-5%) induced higher expression of anti-BPDE-DNA in the group of nonsmokers. Significant relations between the level of anti-BPDE-DNA and PASI score, total duration of the therapy, or time of UVR exposure were not found. Further studies are needed to reduce interpretation uncertainty of this promising bioindicator.

## 1. Introduction

The immune response to the antigenic changes in cancer cells includes expression of serum antibody against these cellular antigens (tumor-associated antigens, TAAs). The serum antibody against TAAs can be used as biomarker in cancer immunodiagnosis. In this case, we can talk about the biomarkers in early secondary prevention [1].

Other specific antibodies indicate the presence of antigenic structures on DNA (DNA adducts) that can play an important role in the process of mutagenesis and/or carcinogenesis. They indicate the presence of increased genotoxic potential (hazard) prior to the formation or development of disease. Here we can talk about the biomarkers in primary prevention. The persistence and stability of given antibodies in the serum is an advantage over other potential markers which are rapidly degraded due to reparation processes (for example chromosomal aberration) [2].

Polycyclic aromatic hydrocarbons (PAHs) are recognized as potential environmental mutagens/carcinogens, requiring bioactivation [3]. Typical representative of the group of PAHs is benzo[a]pyrene (BaP). BaP and its ultimate metabolite benzo[a]pyrene 7,8-diol 9,10-epoxide (BPDE), are classical DNA damaging carcinogens which produce DNA adducts [4]. Formation of DNA adducts is generally one of the assumed mechanisms of PAHs induced mutagenesis/carcinogenesis. In this sense, increased levels of DNA adducts can represent an increased genotoxic potential of exposure. Adducted DNA becomes antigenic and induces immune response by production of antibodies against BPDE-DNA adducts (anti-BPDE-DNA). Anti-BPDE-DNA has been found in serum of PAHs exposed subjects (occupational exposures, smokers) [1, 5]. Accordingly, the presence of circulating anti-carcinogen antibody has been proposed as a biomarker of genotoxic exposure (DNA damage) [6, 7]. However, the use of this bio-indicator is still associated with considerable uncertainty concerning the interpretation of results.

Psoriasis is a chronic, relapsing and remitting immune-mediated inflammatory skin disease that has a prevalence of 2-3% in the world's population, whence 1-2% in Europe [8, 9]. In 1925, William H. Goeckerman from the Mayo Clinic reported the successful use of topical crude coal tar (CCT) and broad-spectrum of UV radiation (UVR) in the treatment of psoriasis [10]. This medical procedure is known as Goeckerman therapy (GT). Despite the availability of newer treatments, classical topical treatments for psoriasis still have an important position for selected patient populations [11]. Topical treatment, including GT, is now applied in approximately 75% of cases which are classified as light to moderately severe forms [12, 13]. Fundamental mechanism of the therapeutic effects of CCT is based on immunosuppression (caused by high portion of PAHs) without evidence of systemic immuno-toxicity [14].

The use of GT has recently decreased for several reasons, including supposed genotoxicity of CCT [14–17]. The CCT is rich in PAHs and GT therefore presents heavy dermal exposure to mutagenic/carcinogenic PAHs. The mutagenicity/carcinogenicity of CCT has been shown in animal studies and studies in occupational settings [18, 19] but there was no clear evidence of an increased risk of skin tumors or internal tumors after the therapy of CCT [3, 20].

Presented study is focused on the serum level of anti-BPDE-DNA in psoriatic patients dermally exposed to PAHs (CCT). General goal is to contribute to better understanding the value of assumed biomarker (anti-BPDE-DNA) for evaluation of the organism's reaction to genotoxic exposure (BaP) and for evaluation of the protective capacity of the immune system (against BPDE-DNA adducts). During the study, we investigated (1) whether changes in the level of genotoxic exposure (CCT/BaP) affect the level of anti-BPDE-DNA and (2) other important factors which could affect exposure the level of anti-BPDE-DNA.

## 2. Material and Methods

### 2.1. Study Groups

The monitored group consisted of patients with chronic stable plague psoriasis, treated by GT at the Clinic of Dermal and Venereal Diseases, University Hospital Hradec Kralove (Czech Republic). Over the period of four years we collected data from 55 adult patients. Of this number 23 patients were treated with dermatological ointment containing 3% of CCT (CCT-3% group) and 32 patients were treated with ointment containing 5% of CCT (CCT-5% group). The CCT-3% group consisted of 12 women and 11 men, the average age of 40 years, age variance 18–75 years, 11 smokers and 12 nonsmokers. The CCT-5% group consisted of 13 women and 19 men, average age of 57 years, age variance 18–75 years, 14 smokers and 18 nonsmokers.

With the use of the questionnaire, we checked the patients for previous excessive exposure to PAHs and artificial UVR. The patients who had prior excessive exposure to PAHs and/or artificial UVR were excluded from the monitored group. The study was approved by the Ethics Committee of the University Hospital in Hradec Kralove, Czech Republic. Informed written consent was obtained from each patient.

### 2.2. Goeckerman Therapy

Dermatological ointment containing 3% and 5% of CCT (pharmaceutical grade crude coal tar; CCT-3% and CCT-5%) was administered daily overnight on psoriatic lesions (10–75% of total body surface in monitored group). The content of BaP in the sample of used pharmaceutical grade crude coal tar was 0.008 mg/g CCT. In morning, the residue of tar ointment was removed from the body (using oil bath) and the patient was whole-body irradiated by UVR. The irradiation was individual according to the disease activity (1–15 min). The density of radiation was 248.17 *μ*W/cm^2^ for UV-B and 132.1 *μ*W/cm^2^ for UV-A (controlled by Sola-Scope 2000 spectrometer; Solatell, UK). Duration of the therapy was determined by the status of the disease and with regard to the treatment efficacy (average duration of 13 days; range of 10–22 days). The efficacy was calculated from the actual state of erythema, desquamation, and skin infiltration by using of PASI score (Psoriasis Area Severity Index) [21]. The therapy was ceased when 50% decrease of PASI was achieved.

### 2.3. Serum Level of Anti-BPDE-DNA

Samples of heparinized venous blood were obtained by venipuncture of the cubital vein before first treatment and again after completion of GT (at the day of dismissal from the hospital) using BD Vacutainer sampling tubes. Blood sera were isolated by centrifugation. Serum samples were stored under −70°C until they were analyzed. Repeated thawing and freezing were avoided. Serum level of anti-BPDE-DNA (IgG/IgM) was analyzed by ELISA method (ELISA-VIDITEST anti-BPDE-DNA human, VIDIA, Jesenice, Czech Republic). The results were expressed in the form of Evaluation Index (EI = absorbance of evaluated serum/absorbance of high positive control serum). Samples with EI less than 0.5 are termed as the serum with low levels of anti-BPDE-DNA. Analogously, serum samples with EI greater than 0.5 are referred to as the serum with high level of anti-BPDE-DNA (ELISA-VIDITEST anti-BPDE-DNA human, VIDIA, Jesenice, Czech Republic).

### 2.4. Statistical Analysis

Data were analyzed by using MATLAB rel. 2013b software (Mathworks, Inc., Massachusetts, USA). Because the Lilliefors test of normality had rejected the hypothesis of normal distribution, nonparametric tests were used. Data were analyzed by the Wilcoxon signed rank test. The effect of smoking was evaluated by the Wilcoxon rank sum test. The association between serum level of anti-BPDE-DNA after the therapy and selected parameters was evaluated by Spearman Rank Order Correlations.

## 3. Results

**Figure 1:**
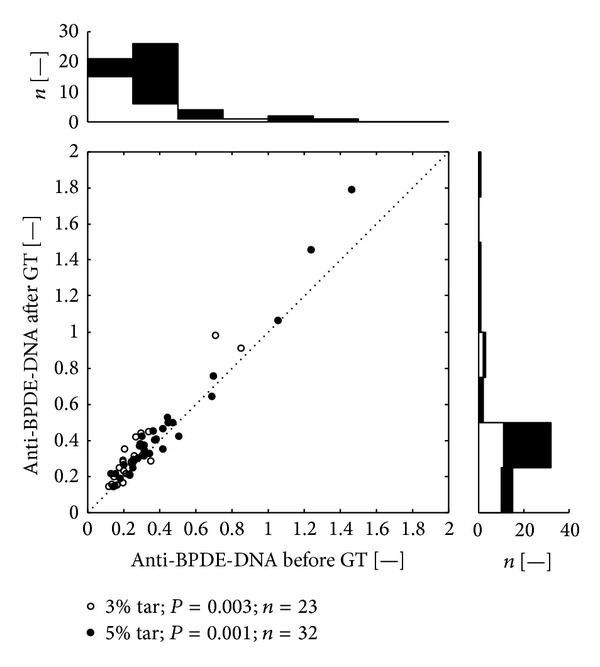
Distribution of values of anti-BPDE-DNA (all patients). Legend: Scatter plot depicts anti-BPDE-DNA values before and after the GT therapy. All together 55 dots represent 110 measurements, each dot belongs to a one patient.

After the statistical processing of results stated in Tables 1–3, we have found that both variants of the therapy (group with CCT-3% and group with CCT-5%) significantly increased serum level of anti-BPDE-DNA. The therapy with CCT-5% increases the level of anti-BPDE-DNA at a higher level of significance (*P* < 0.001) than the therapy with CCT-3% (*P* < 0.01). However, it must be stated, that majority of anti-BPDE-DNA values (approx. 85% of all samples) ranged in the field of low values (EI < 0.5), regardless whether it was collected before or after therapy (Figure 1).

Both variants of the therapy significantly improved the status of the disease (decreased PASI score; *P* < 0.001) which generally confirmed high effectiveness of the therapy. In comparison to CCT-3% variant, the effectiveness of the CCT-5% variant, expressed as PASI score, was higher by about 12% (Table 4).

**Figure 2:**
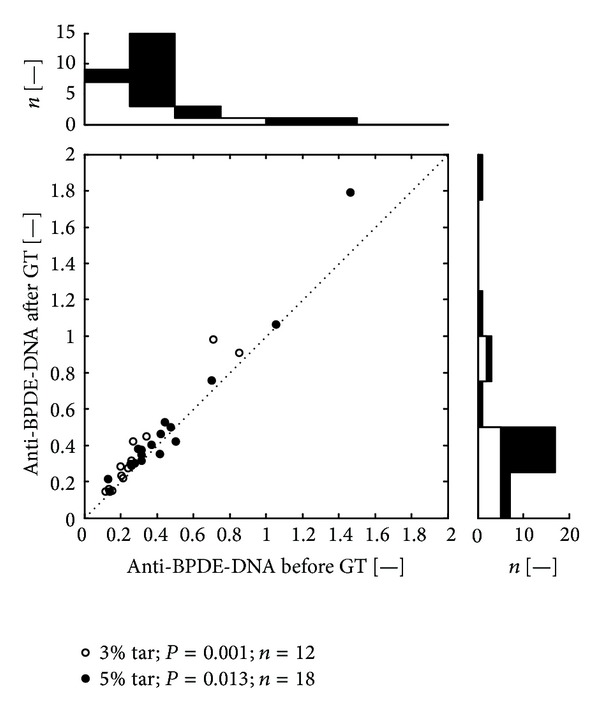
Distribution of values of anti-BPDE-DNA (non-smokers). Legend: Scatter plot depicts anti-BPDE-DNA values before and after the GT therapy. All together 30 dots represent 60 measurements, each dot belongs to a one patient.

**Figure 3:**
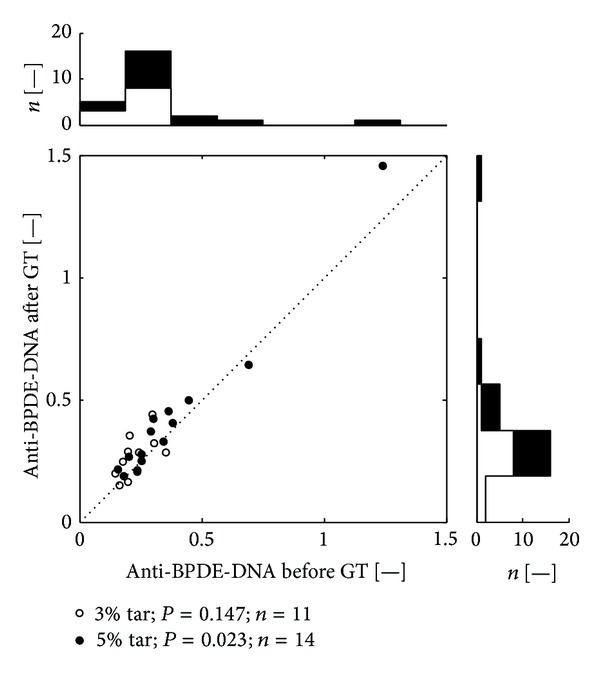
Distribution of values of anti-BPDE-DNA (smokers). Legend: Scatter plot depicts anti-BPDE-DNA values before and after the GT therapy. All together 25 dots represent 50 measurements, each dot belongs to a one patient.

The serum levels of anti-BPDE-DNA in smokers and non-smokers (before and after the treatment) were comparable (Tables 2 and 3). However, it seems that both variants of the treatment induced higher expression of anti-BPDE-DNA in the group of non-smokers (Tables 2 and 3 and Figures 2 and 3).

The therapy increased the level of anti-BPDE-DNA and decreased PASI score; however, we found no significant relationship between these two parameters (CCT-3%, *r* = −0.17; CCT-5%, *r* = −0.22). As well, the relationships between final level of anti-BPDE-DNA and total duration of the therapy (CCT-3%, *r* = 0.21; CCT-5%, *r* = 0.10), and between final level of anti-BPDE-DNA and time of UVR exposure (CCT-3%, *r* = 0.18; CCT-5%, *r* = 0.25) were not significant.

Scatter plots (Figures 1, 2, and 3) depicts anti-BPDE-DNA values before and after the GT therapy. Each dot belongs to one patient. The open dots mark patients from the group treated by CCT-3%, the black filled dots mark patients treated by CCT-5%. Values above the dotted diagonal line reflect increase of the anti-BPDE-DNA after the therapy. Probability of difference between the pre- and the post-treatment medians was evaluated by Wilcoxon signed rank test. The top histogram shows distribution of the anti-BPDE-DNA before treatment; the white color corresponds to the patients with CCT-3%, added black bars represent the patients with CCT-5%. The right side histogram corresponds to the after treatment distribution of anti-BPDE-DNA values.

## 4. Discussion

After exposure to BaP, antibodies (IgG and IgM) against BaP can be detected in human serum. These antibodies are capable of scavenging BaP from the environment and thereby block its metabolic activation in cells [1]. This immune response may decrease the risk of cancer.

The situation is different with antibodies (IgG and IgM) against BPDE-DNA adducts. Elevated levels of these antibodies were found in the population of individuals exposed to higher concentrations of PAHs and corresponded with increased levels of DNA adducts at a group level. The serum level of anti-BPDE-DNA reflects either the previous exposure of the individual to BaP and/or the immune status and protective capacity of the immune system against BaP induced cancer [5].

Biological monitoring includes indicators of exposure, effect and susceptibility. BPDE-DNA adducts can be indicative of both exposure and genotoxic effects of BaP. Circulating anti-BPDE-DNA signals the presence of BPDE-DNA adducts. The intensity of that signal depends (among others) on the level of BPDE-DNA adducts. The level of BPDE-DNA adducts is determined by the character of BaP metabolism and by the degree of adaptation and reparation processes.

There are only few epidemiological studies of PAHs exposed population to assess the impact of carcinogen-specific antibodies on their risk of tumor development and on their relation to other indicators of genotoxic exposure [1, 5]. Also, few attempts have been made in vivo or in vitro to understand the implications of an antibody response to metabolic activation of carcinogens and carcinogenesis. Recent study provided evidence that specific humoral immunity may modulate the genotoxic effect induced by subsequent carcinogen exposure, however, the mechanisms involved remain largely unexplored [1].

Scientific data concerning the level of anti-BPDE in the treatment of CCT are very limited. In one study, a group of psoriatic patients (treated by CCT) was used as a model for evaluation the suitability of immunoassays for the biomonitoring of exposure to BaP and related PAHs. The assays included measurement of PAH diol epoxide-DNA adducts in white blood cells, PAH-albumin adducts and serum levels of antibodies recognizing BP diol epoxide-DNA adducts. PAH-DNA adducts were elevated in patients: mean 6.77 ± 12.05/10(8) compared to controls: 4.90 ± 8.81/10(8), (*P* = 0.12), however there was no difference in PAH-albumin adducts between patients (mean 0.61 ± 0.31 fmol/micrograms) and controls (0.63 ± 0.30 fmol/micrograms). About 30% of patients and controls had measurable titer of antibodies recognizing BPDE-DNA adducts [22].

In presented study we have found that both variants of the therapy (group with CCT-3% and group with CCT-5%) significantly increased serum level of anti-BPDE-DNA (Table 1, Figure 1). Nevertheless, it must be stressed that majority of anti-BPDE-DNA values (85% of all samples) ranged within the field of low values (EI < 0.5).

A person is exposed to PAHs from many sources daily. These substances are virtually ubiquitous and contaminate air, water and food [2, 14]. From this perspective, it is understood that a certain level of antibodies will be present in all individuals. Smoking increases the level of BPDE-DNA adducts [23]. Serum anti-BPDE-DNA can be detected in smokers, and its persistence for months after smoking cessation suggests its usefulness for relatively long-term surveys [24]. On the other hand, it seems that long term chronic exposure to PAHs (for instance, by smoking) reduces body immune response and presumably reduces levels of anti-PAHs antibodies [1, 25].

Similar situation can be seen at GT. Application of CCT (PAHs) increases the level of BPDE-DNA adducts and consequently increases the production of anti-BPDE-DNA. However, PAHs have also immunosuppressive effect and can reduce the production of anti-BPDE-DNA in this way. Therefore, the final level of anti-BPDE-DNA is probably the result of these conflicting processes.

Before the therapy we found in the group of smokers lower levels of anti-BPDE-DNA. It may be related to the above-described immunosuppressive effect of chronic exposure to tobacco smoke which can reduce expression of anti-BPDE-DNA. This assumption is also supported by the fact that in non-smokers, we found more significant increase of the anti-BPDE-DNA level than in smokers (Tables 2 and 3 and Figures 2 and 3). In addition, adaptation mechanisms (in smokers) will undoubtedly reduce the answer of the organism per unit dose of mutagenic stimulus (BaP).

State of repair mechanisms can significantly affect the level of BPDE-DNA adducts (and consequently anti-BPDE-DNA). Smoking may induce the repair mechanism but also it can cause their overload.

GT is used mainly for chronic stable plaque psoriasis and has a high benefit for patients with refractory psoriasis [13]. Also, in the present study, both variants of the therapy significantly improved the status of the disease (decrease of PASI score; *P* < 0.001) and confirmed generally high effectiveness of the GT.

## 5. Conclusion

The serum level of anti-BPDE-DNA in psoriatic patients (dermally exposed to different level of CCT/PAHs) has been observed. Elevated level of genotoxic exposure (BaP) resulted in an increased level of anti-BPDE-DNA. However, almost all levels of anti-BPDE-DNA ranged in the field of low values. Both variants of GT (CCT-3% a CCT-5%) induced higher expression of anti-BPDE-DNA in the group of non-smokers. Significant relationships between the level of anti-BPDE-DNA and PASI score, total duration of the therapy or time of UVR exposure were not found. Further studies are needed to reduce interpretation uncertainty of this promising bio-indicator.

## Figures and Tables

**Table 1:** The serum level of anti-BPDE-DNA before and after the therapy (all patients).

CCT content	Anti-BPDE-DNA		Statistical significance of differences
	Before GT	After GT	
CCT-3% (*n* = 23)	0.22 (0.18–0.29)	0.28 (0.20–0.34)	*P* < 0.01
CCT-5% (*n* = 32)	0.31 (0.25–0.45)	0.37 (0.28–0.48)	*P* < 0.001
Statistical significance of differences	*P* < 0.01	*P* < 0.05	

Anti-BPDE-DNA values (expressed as the EI) are presented as median (lower–upper quartile) because of nonnormal data distribution; *n*: number of subjects; *P*: Wilcoxon matched-pairs test.

**Table 2:** The serum level of anti-BPDE-DNA before and after the therapy (nonsmokers).

CCT content	Anti-BPDE-DNA		Statistical significance of differences
	Before GT	After GT	
CCT-3% (*n* = 12)	0.23 (0.17–0.30)	0.28 (0.19–0.43)	*P* < 0.001
CCT-5% (*n* = 18)	0.34 (0.28–0.47)	0.38 (0.30–0.50)	*P* < 0.05
Statistical significance of differences	*P* < 0.05	**NS**	

Anti-BPDE-DNA values (expressed as the EI) are presented as median (lower–upper quartile) because of nonnormal data distribution; *n*: number of subjects; *P*: Wilcoxon matched-pairs test.

**Table 3:** The serum level of anti-BPDE-DNA before and after the therapy (smokers).

CCT content	Anti-BPDE-DNA		Statistical significance of differences
	Before GT	After GT	
CCT-3% (*n* = 11)	0.21 (0.18–0.28)	0.28 (0.20–0.31)	**NS**
CCT-5% (*n* = 14)	0.30 (0.24–0.38)	0.35 (0.25–0.45)	*P* < 0.05
Statistical significance of differences	**NS**	**NS**	

Anti-BPDE-DNA values (expressed as the EI) are presented as median (lower–upper quartile) because of nonnormal data distribution; *n*: number of subjects; *P*: Wilcoxon matched-pairs test.

**Table 4:** The effectiveness of the therapy (PASI score).

CCT content	PASI score		Statistical significance of differences	Efficiency (%)
	Before GT	After GT		
CCT-3% (*n* = 23)	16.20 (12.70–20.85)	7.80 (6.60–10.70)	*P* < 0.001	50.9 (41.9–60.58)
CCT-5% (*n* = 32)	17.10 (14.80–20.60)	6.05 (4.30–7.95)	*P* < 0.001	62.7 (52.4–74.0)
Statistical significance of differences	**NS**	*P* < 0.05		*P* < 0.01

The effectiveness of the therapy is expressed as the PASI score; the values are presented as median (lower–upper quartile) because of nonnormal data distribution; *n*: number of subjects; *P*: Wilcoxon matched-pairs test.

Calculation of the efficiency of the treatment: (100 − [PASI after GT/PASI before GT] × 100).
